# Recommendations, guidelines, and best practice for the use of human induced pluripotent stem cells for neuropharmacological studies of neuropsychiatric disorders

**DOI:** 10.1016/j.nsa.2023.101125

**Published:** 2023-03-20

**Authors:** Lucia Dutan Polit, Ilse Eidhof, Rhiannon V. McNeill, Katherine M. Warre-Cornish, Cristine Marie Yde Ohki, Natalie Monet Walter, Carlo Sala, Chiara Verpelli, Franziska Radtke, Silvana Galderisi, Armida Mucci, Ginetta Collo, Frank Edenhofer, Maija L. Castrén, János M. Réthelyi, Morten Ejlersen, Sonja Simone Hohmann, Mirolyuba S. Ilieva, Renate Lukjanska, Rugile Matuleviciute, Tanja Maria Michel, Femke M.S. de Vrij, Steven A. Kushner, Bas Lendemeijer, Sarah Kittel-Schneider, Georg C. Ziegler, Doris Gruber-Schoffnegger, R. Jeroen Pasterkamp, Amal Kasri, Marie-Claude Potier, Jürgen A. Knoblich, Oliver Brüstle, Michael Peitz, Emilio Merlo Pich, Adrian J. Harwood, Elsa Abranches, Anna Falk, Anthony C. Vernon, Edna Grünblatt, Deepak P. Srivastava

**Affiliations:** a Department of Basic and Clinical Neuroscience, Institute of Psychiatry, Psychology and Neuroscience, King's College London, London, United Kingdom; b MRC Centre for Neurodevelopmental Disorders, King's College London, London, United Kingdom; c Department of Neuroscience, Karolinska Institutet, Stockholm, Sweden; d Department of Psychiatry, Psychotherapy and Psychosomatic Medicine, University Hospital, University of Würzburg, D-97080, Würzburg, Germany; e National Institute for Biological Standards and Control, South Mimms, UK; f Department of Child and Adolescent Psychiatry and Psychotherapy, Psychiatric University Hospital Zurich (PUK), University of Zurich, Zürich, Switzerland; g CNR Neuroscience Institute Milano, Via Raoul Follereau 3, 20854, Vedano al Lambro, MB, Italy; h Department of Child and Adolescent Psychiatry, University Hospital, University of Wuerzburg, D-97080, Würzburg, Germany; i Department of Mental and Physical Health and Preventive Medicine, University of Campania Luigi Vanvitelli, Naples, Italy; j Department of Molecular and Translational Medicine, University of Brescia, Viale Europa 11, 25123, Brescia, Italy; k Department of Molecular Biology & CMBI, Genomics, Stem Cell Biology & Regenerative Medicine Group, Leopold-Franzens-University Innsbruck, Austria; l Medicum, Department of Physiology, University of Helsinki, Helsinki, Finland; m Department of Psychiatry and Psychotherapy, Semmelweis University, Budapest, Hungary; n Department of Psychiatry, Department of Clinical Research, University of Southern Denmark and Psychiatry in the Region of Southern Denmark, Odense University Hospital, Odense, Denmark; o Department of Psychiatry, Erasmus University Medical Center, Rotterdam, the Netherlands; p Department of Translational Neuroscience, Evotec SE, Essener Bogen 7, D-22419 Hamburg, Germany; q Department of Translational Neuroscience, University Medical Center Utrecht Brain Center, Utrecht University, Universiteitsweg 100, 3584 CG, Utrecht, the Netherlands; r ICM Paris Brain Institute, CNRS UMR7225, INSERM U1127, Sorbonne University, Hôpital de la Pitié-Salpêtrière, 47 Bd de l’Hôpital, 75013, Paris, France; s IMBA - Institute of Molecular Biotechnology of the Austrian Academy of Sciences, Vienna Biocenter (VBC), Vienna, Austria; t Medical University of Vienna, Department of Neurology, Vienna, Austria; u Institute of Reconstructive Neurobiology, University of Bonn Medical Faculty & University Hospital Bonn, Germany; v Cell Programming Core Facility, University of Bonn Medical Faculty, Bonn, Germany; w Gelf Health, Milano, Italy; x Neuroscience and Mental Health Innovation Institute (NMHII) and School of Biosciences, Cardiff University, UK; y Department of Experimental Medical Science, Lund Stem Cell Center, Lund University, Lund, Sweden; z Neuroscience Center Zurich, University of Zurich and the ETH Zurich, Zürich, Switzerland; aa Zurich Center for Integrative Human Physiology, University of Zurich, Zürich, Switzerland

**Keywords:** Neuropsychopharmacology, Cellular models, Neuropsychiatry, Drug discovery, Neurodevelopmental disorders, Sex as a biological variable (SABV)

## Abstract

The number of individuals suffering from neuropsychiatric disorders (NPDs) has increased worldwide, with 3 million disability-adjusted life-years calculated in 2019. Though research using various approaches including genetics, imaging, clinical and animal models has advanced our knowledge regarding NPDs, we still lack basic knowledge regarding the underlying pathophysiological mechanisms. Moreover, there is an urgent need for highly effective therapeutics for NPDs. Human induced pluripotent stem cells (hiPSCs) generated from somatic cells enabled scientists to create brain cells in a patient-specific manner. However, there are challenges to the use of hiPSCs that need to be addressed. In the current paper, consideration of best practices for neuropharmacological and neuropsychiatric research using hiPSCs will be discussed. Specifically, we provide recommendations for best practice in patient recruitment, including collecting demographic, clinical, medical (before and after treatment and response), diagnostic (including scales) and genetic data from the donors. We highlight considerations regarding donor genetics and sex, in addition to discussing biological and technical replicates. Furthermore, we present our views on selecting control groups/lines, experimental designs, and considerations for conducting neuropharmacological studies using hiPSC-based models in the context of NPDs. In doing so, we explore key issues in the field concerning reproducibility, statistical analysis, and how to translate in vitro studies into clinically relevant observations. The aim of this article is to provide a key resource for hiPSC researchers to perform robust and reproducible neuropharmacological studies, with the ultimate aim of improving identification and clinical translation of novel therapeutic drugs for NPDs.

## Highlights

• hiPSC-based models are a powerful tool in neuropharmacological research.
• Careful experimental design is essential to produce robust and reproducible data.
• Recommendations for best practice are provided to improve research standardisation.

## 1 Introduction

Throughout the past decades the global prevalence of neuropsychiatric disorders (NPDs) has steadily increased and represents a major societal challenge (Collaborators, 2022). In 2019, NPDs were the cause of 3 million disability-adjusted life-years (DALYS) worldwide (Collaborators, 2022), with an estimated total cost of more than 4% of GDP in Europe alone (Union, 2018). Current available therapies for NPDs can be effective in the treatment of symptoms, although 20–60% patients are treatment-resistant or difficult to treat (Collaborators, 2022). Moreover, these drugs do not address the fundamental pathophysiological causes, which remain elusive. The development of novel, highly effective therapeutics is an urgent unmet medical need, however delivering these will first require a deeper understanding of how the complex genetic landscape associated with these disorders and the underlying pathophysiological mechanisms contribute to NPDs.

The complexity and heterogeneity of NPDs present significant hurdles to meet this challenge. Compounding this, many of our insights into the underlying pathophysiology to date arose from animal models. This is mainly because it is both not possible and unethical to conduct detailed investigations into the neurobiology or underlying molecular mechanisms of NPDs in living patients. Despite the enormous progress animal models have provided in our understanding of the aetiology of NPDs, it is important to note that these models are unable to fully recapitulate the complex genetic background, the varying environmental effects and the pathophysiology of psychiatric disorders (Anderson et al., 2021; Ardhanareeswaran et al., 2017; Singh and Seed, 2021; Stanford, 2020; Stock et al., 2021). Additionally, and perhaps most importantly, these models do not fully recapitulate human physiology and human neurodevelopment (Anderson et al., 2021; Ardhanareeswaran et al., 2017; Pasteuning-Vuhman et al., 2021; Stock et al., 2021). This is exemplified by the low percentage of preclinical studies that result in novel and viable drug targets (Singh and Seed, 2021; Stanford, 2020), although it must be noted that this lack of translation may be due to incorrect use of animal models and poorly designed clinical trials (Bale et al., 2019). Consequently, there is an increasing interest and acknowledgement that complementary human-based models are needed to study NPDs within a cellular context that better recapitulates human physiology, and that can capture the complex genetic landscape thought to contribute to NPDs.

Since the discovery of embryonic stem (ES) cells in the early 1990's, and the demonstration of the ability to generate human induced pluripotent stem cells (hiPSCs) from adult somatic cells by Yamanaka and Takahashi in 2006 and 2007 (Takahashi et al., 2007; Takahashi and Yamanaka, 2006), pluripotent stem cells (PSCs) have become a major tool in several research areas. These cells have the ability to differentiate into cell types from all three germ layers in vitro, and thus have great potential for discovery and translational science, human neurodevelopment, and increasingly as a platform for drug development. In this review, we will use PSCs as an umbrella term to refer to both human ES cells (hESCs) and hiPSCs, or use these terms when discussing either cell type specifically.

A major attraction of using PSCs generated from somatic cells is that they retain the genetic background of the host donor. This is of particular interest in the case of hiPSCs generated from individuals with specific disorders, including NPDs. These patient-derived hiPSCs provide a unique in vitro system that specifically and faithfully replicate the host's genetic background in a dish. This greatly aids the study of underlying pathophysiology of disorders with complex genetic backgrounds. It is also key for the development of potential personalised medicine approaches, and for drug discovery/safety pharmacology approaches, where genetic heterogeneity plays an important role.

Despite the promise of PSCs, there are specific challenges and considerations that need to be taken into account when conducting studies using these cells. Stem cell banks, registries and consortia have crucial roles to play in promoting the quality and reproducibility of stem cell research. An important aspect of this work is the development of quality control guidelines and written standards. Key examples include guidelines published by the International Stem Cell Banking Initiative (ISCBI) (Andrews et al. 2015; International Stem Cell Banking Initiative, 2009); the International Society for Stem Cell Research (ISSCR) (https://www.isscr.org/guidelines) and the Global Alliance for iPSC Technologies (GAiT) (Sullivan et al., 2018). Excellent reviews have been published on topics such as Good Cell Culture Practice (GCCP) (Pamies et al., 2018, 2022); generation of hiPSCs, including Good Manufacturing Practice (GMP) grade cells (Abranches et al., 2020); as well as guidelines and recommendations for biobanking and molecular and genetic studies using hiPSC lines (Anderson et al., 2021; Bock et al., 2011; Daley et al., 2016; De Los Angeles et al., 2021; Engle et al., 2018; Hoffman et al., 2019; Lister et al., 2011; Lovell-Badge et al., 2021; Steeg et al., 2021; Yaffe et al., 2016). Stem cell registries have built on these recommendations, to provide a searchable platform where users can find information on the provenance, traceability, cell culture methodology and quality control information for registered PSC lines. The largest example of these is European Human Pluripotent Stem Cell Registry (hPSC reg), which is home to information on over 4000 hPSC lines (https://hpscreg.eu/; Seltmann et al., 2016; Isasi et al., 2014). Physical standards and reference reagents have also been proposed as tools to harmonise PSC research. A recent study compared genetic, genomic and phenotypic properties of candidate hiPSC lines and proposed a high performing line as a standard, accessible to the stem cell community (Pantazis et al., 2022).

Access to these resources provides a sound basis for ethical and quality assurance of hiPSC research. However, there remain several key areas of consideration that have not been reviewed in depth, but that are of critical importance for studies where hiPSCs are generated from patients with NPDs. In this paper, we therefore aim to provide an integrated view of best practice for neuropharmacological and neuropsychiatric research using hiPSCs. Specifically, we outline considerations pertaining to patient recruitment, experimental design and reproducibility, the use of hiPSC-derived cells for neuropharmacological studies, and the (forward) translation of in vitro studies for clinical relevance. Guidance on these key issues is intended to support accuracy, reproducibility, and clinical utility in neuropsychiatric research. A summary of our key recommendations can be found in Box 1.

Box 1 **Summary of Key Recommendations**

1) Where possible, generation/use of patient-specific hiPSC lines with detailed demographic, clinical, medical, diagnostic and genetic data (incl. polygenic risk scores), along with familial history and non-clinical measures to provide context to sources of variation between donor lines.
2) Careful consideration of donor genetic background, including donor sex as an important biological variable.
3) Prioritise increasing overall donor number (biological replicates) instead of multiple clones per donor (technical replicates), in order to increase statistical power – if technically feasible, ideally with the use of >1 clone from each line.
4) Careful consideration when selecting control hiPSC lines; we recommend the use of sex-matched family members (for studying specific genetic variation, which must be confirmed present in the patient and absent in the family member), or age-, sex- and ethnicity-matched individuals from a similar geographical location. We also acknowledge the utility of gene editing approaches, especially in the study of specific genetic loci.
5) When carrying out pharmacological studies, we recommend identifying the relevant end-point assays to characterise time- and concentration (dose-)response curves before progressing into agonist/antagonist studies and to apply the appropriate statistical study design and analysis.

Alt-text: Box 1

## 2 Donor information, selection, and consent

In order to conduct robust and representative hiPSC-based studies, basic patient demographics need to be taken into account (e.g. ethnicity, age, and sex), and should be as balanced as possible between cohorts. Due to the heterogeneity of NPDs, we suggest that data collection should be extended as much as possible beyond these basic demographic factors, ensuring strict adherence to data protection laws. Patient donors should be carefully selected based on these data, in order to increase the likelihood of discovering robust and specific cellular phenotypes by reducing data variability. Another challenge is the selection of control hiPSC lines. In this context, “control” hiPSC lines are often broadly defined as individuals who are “apparently healthy” or “neurotypical” (lack of a diagnosis). We suggest that the selection of appropriate control lines is as important as selection of patient lines. Below, we explore multiple donor-specific factors that should be determined and taken into account when generating/selecting hiPSCs for experiments, from both “patient” and “control” cohorts.

### 2.1 Choosing suitable donors from patient cohorts – going beyond diagnosis-directed hiPSC generation

Early studies using hiPSCs in the context of NPDs have tended to focus on a specific diagnosis. A wider range of patient/donor characterisation is now acknowledged as a valuable tool (Campo-Arias et al., 2021; Waszczuk et al., 2020). This is because diagnostic categories such as ICD-10/ICD-11 and DSM-5 might not consider the overlap between diagnoses on a pathogenetic level (Barkhuizen et al., 2020; Regier, 2007). For example, it has been suggested that similar neurobiological processes or shared mechanisms might be causative for autism and schizophrenia (Chisholm et al., 2015), mood disorders and schizophrenia (Baumann and Bogerts, 1999), restrictive food disorder and eating disorders (Becker et al., 2020), post-traumatic stress disorder (PTSD) and mood symptoms (Carmassi et al., 2020), and for depression and neurodegenerative diseases (Hussain et al., 2020). Neurobiological correlates for these similarities have been found in brain morphometry (Madre et al., 2020) and cell-based models (Enwright and Lewis, 2021; Glausier et al., 2020), and are in accordance with results from genome wide association studies (GWAS) showing an overlap in risk genes (Muntane et al., 2021; Nenadic et al., 2020; Ni et al., 2021) as well as cell-type-specific expression changes (Bryois et al., 2022; Cameron et al., 2022). It has therefore been suggested that analysing a spectrum of traits (e.g. phenotypes) might be better suited to identifying pathomechanisms (Marin et al., 2020; Mottron and Bzdok, 2020). We recommend psychometric testing of all donors, both healthy controls and individuals with NPDs. For example, a minimum of a high validity intelligence testing (e.g. via WISC or WAIS) and a reliable battery of (self-) assessment instruments scaling mood, anxiety, social behaviour, cognitive, attention-deficit/hyperactivity disorder (ADHD)-like symptoms and personality traits should be performed. Additionally, disorder-specific rating tools should also be implemented (e.g. Adult-ADHD-Self-Report-Scale for ADHD (Kessler et al., 2005)) in order to obtain in-depth clinical phenotypes. Important psychopathological parameters such as subtype of the NPD, age of onset, treatment response, number and duration of episodes in recurring disease, current psychotropic and other medication are also vital. Donors can therefore be selected based on objective phenotypic measurements, rather than a dichotomous diagnosis.

Where possible, non-psychiatric medical history should also be collected. It has now been demonstrated that hiPSCs show epigenetic memory to some extent, and morbidity as well as medical treatment could therefore potentially alter cellular morphology and function (Bar-Nur et al., 2011; Efrat, 2020). However, it should be noted that with the development of more accurate models of epigenetic age, there is an increasing appreciation that hiPSC and differentiated cells have a foetal epigenetic age (Steg et al., 2021). Nevertheless, highly prevalent diseases such as obesity and diabetes have been shown associated with considerable epigenetic changes (Rosen et al., 2018). Collecting medical history also allows the researcher to gain information regarding environmental factors present over the lifetime, which could be extraneous variables. For example, recreational drug and nicotine use can lead to an altered epigenome, which was found to be maintained throughout reprogramming into hiPSCs (Mackey et al., 2018). Likewise, adverse perinatal life events such as viral infections, hypoxia or stress can be seen in the epigenome (Lux, 2018). Moreover, environmentally induced changes to the epigenome have been found conserved over several generations, both for alcohol and drug abuse (Pandey et al., 2017; Wimmer et al., 2017).

### 2.2 Donor genotype

Researchers often focus on a specific genetic loci/target of interest, in which variation(s) has been associated with the development of a particular NPD. However, as the genome is highly donor-specific, it is essential to try and control for the effects of variability in the rest of the genome. To control for the genetic heterogeneity we recommend to use non-affected (sex-matched) family members for the generation of control hiPSC lines. The rationale for this is that the genetic backgrounds in the control and patient hiPSC lines should be similar, especially compared to the use of non-related individuals. However, it should be noted that family members may also carry increased genetic burdens/variants associated with NPDs. When sex and age matched family members are not available or if these members carry genetic risk factors of NPDs, these risk alleles can contribute to the atypical phenotype and will therefore not be suitable as “controls”. In such cases, we recommend the use of non-related, age and sex-matched individuals with the same/similar ethnicities as donors for the generation of control hiPSC lines. In very specific cases where patients have mutated as well as non-mutated cells (somatic mosaicism), hiPSC clones may be obtained that are fully isogenic except for the disease-causing mutation. For example, isogenic hiPSC clones were generated from an individual with Down syndrome carrying a partial trisomy 21 (Murray et al., 2015).

Genome editing techniques are also an alternative to standardise genetic backgrounds via the generation of isogenic cell lines. Cas9-mediated genome editing can be used to reverse genomic rearrangements to obtain edited hiPSC clones with an identical genetic background to the parental line, thus reducing genetic heterogeneity and phenotypic variability (Bassett, 2017). This approach has been utilised with great success in the study of specific mutations in the context of NPDs (Gonzalez, 2016; Hazelbaker et al., 2017; Ikeda et al., 2018; Schrode et al., 2019). Other alternative methods proposed have been to reverse CNVs by producing microdeletions or microduplications with single-guide targeting repetitive elements in the CNV (Tai et al., 2016). There is emerging evidence that targeting CNV-flanking regions by CRISPR/Cas9 can generate not only deletions but can also result in duplication variants in rare instances. This has been demonstrated in hiPSCs for 15q13.3 and 16p11.2 variants larger than 500 ​kb in size (Tai et al., 2016). Furthermore, transposase-associated CRISPR is a promising new tool to generate site-directed insertions (Klompe et al., 2019; Strecker et al., 2019), which could facilitate the generation of isogenic duplication lines. More recently, the combined use of CRISPR-activation and inhibition has been used to study the polygenic nature of NPDs. For example, this approach has been used to demonstrate that increased or decreased expression of specific genes associated with schizophrenia may act in a synergistic manner (Schrode et al., 2019). This has provided insight into the complex way multiple genetic factors may interact to impact cell physiology. Overall, CRISPR-Cas9 genetic editing is currently regarded as the gold standard for controlling background genetic variation. However, despite the rapid development of this area and application for use with PSCs, there are currently some limitations associated with genome editing approaches that may be of consideration when studying NPDs. For example, it is currently not possible to use genome editing to comprehensively study complex genetic variations associated with NPDs. In the case of schizophrenia, over 250 single nucleotide polymorphisms (SNPs) have been associated with the disorder (Trubetskoy et al., 2022). However, whether these SNPs are directly relevant and drive pathophysiology or are simply in linkage disequilibrium with causal variants remains unclear. Therefore, systematically studying variants identified from GWAS studies, either by themselves or in combination with other potential causative variants may have limited benefit in understanding pathogenic mechanisms associated with specific disorders (De Los Angeles et al., 2021). At a more practical level, gene-editing approaches can be both costly and time-consuming.

Regardless of the approach used, comprehensive genetic analysis is recommended for all hiPSC lines. As a minimum, we suggest assessment of hiPSC genome by high density SNP array (for virtual karyotyping and genotyping) or, ideally, whole genome sequencing. Moreover, such analysis should be performed both in hiPSCs and the somatic parental cells from which they were derived. On a basic level, this ensures that the reprogramming process did not change the genetic variant of interest. Several lines of evidence indicate that PSCs may accumulate mutational load upon long-term culturing (D'Antonio et al., 2018; Halliwell et al., 2020; Kuijk et al., 2020), and assessment of genome integrity may aid in identifying PSC lines that have accumulated mutational loads. There is also evidence to suggest that genetic background may be an important factor in driving variability between PSC lines, including in their ability to differentiate into specific cell fates (Bock et al., 2011; Cuomo et al., 2020; Jerber et al., 2021). Genetic analysis also allows the construction of polygenic risk scores (PRS) (Bonder et al., 2021; Danecek et al., 2016; Volpato and Webber, 2020), permitting the stratification and classification of hiPSC lines to aid in reducing heterogeneity or selection of lines with defined genetic burdens (Bonder et al., 2021; Coleman, 2022; Dobrindt et al., 2021; Hoekstra et al., 2017; Jerber et al., 2021). These scores could be a further factor in donor selection. For example, the use of hiPSCs from control donors with a low PRS for a specific NPD could be used in conjunction with hiPSCs from affected donors with a high PRS for the NPD, potentially increasing the likelihood of being able to identify disease-associated altered cellular phenotypes (Bhat et al., 2022; Coleman, 2022; Dobrindt et al., 2021; Page et al., 2022).

Another benefit of performing in-depth genetic analysis is that any alterations to the genome due to reprogramming can be identified and considered when selecting donors and designing experiments (Bock et al., 2011; Bonder et al., 2021; Halliwell et al., 2020; Hoekstra et al., 2017; Volpato and Webber, 2020). Up to 70% hiPSC lines develop *de novo* CNVs during the reprogramming and expansion process, therefore it is essential that these CNVs be identified, in case they are located in a gene important for the experiment (Bonder et al., 2021; Cuomo et al., 2020; D'Antonio et al., 2018; Halliwell et al., 2020; Kuijk et al., 2020). Assessment of PRS and examination of the presence of CNVs or other genetic variants within the genome is also critical for control hiPSC lines (Coleman, 2022; Dobrindt et al., 2021; Hoekstra et al., 2017; Volpato and Webber, 2020). Knowledge of control line genetic background would help facilitate the selection of appropriate control lines for specific studies. It is also of note that others have recommended the use of common “Rosetta-stone” control hiPSC lines as an approach to reduce variability across studies and centres (Volpato and Webber, 2020). Consistent with this concept, a recent study has identified and proposed the KOLF2.1J line as a reference hiPSC line for collaborative studies (Pantazis et al., 2022).

### 2.3 Treatment responsive vs non-responsive patients

The neurobiological mechanisms of response and non-response to specific psychotropic drug treatments remain unknown, and more insight is crucial to develop predictive biomarkers aiming at personalised or precision medicine approaches, as well as to screen for novel medications. The differences in treatment response are likely to be at least partly genetic and could thereby increase data heterogeneity. Recent studies using patient-derived hiPSCs were able to recapitulate the responder/non-responder status of the donor in vitro (Akkouh et al., 2022; Bardy et al., 2020; Collo et al., 2020; Hribkova et al., 2022; Mertens et al., 2016; Stern et al., 2018). Lithium responsiveness in bipolar patients appears to have a strong genetic influence, which makes it a promising endophenotype for hiPSC-based research (Schubert et al., 2021; Stone et al., 2021). Previous studies have shown that hiPSC-differentiated neuronal cells from bipolar patients show an increased excitability compared with healthy control cells, which was ameliorated by in vitro lithium treatment only in responders and not in non-responders (Mertens et al., 2016; Stern et al., 2018). A recent study demonstrated improvement of mitochondrial respiration after in vitro lithium treatment only in neuronal progenitor cells of lithium responders (Osete et al., 2021). In the context of major depression, there are two studies investigating hiPSC-derived forebrain neurons from patients responsive to selective-serotonin-reuptake-inhibitors (SSRI) compared to non-responsive patients (Vadodaria et al., 2019a, 2019b). Here it could be shown that after 5-Hydroxytryptamine (serotonin, 5-HT) treatment in vitro*,* non-responder neuronal cells had significantly higher calcium activity compared to responder and healthy controls due to upregulated 5-HT2A and 5-HT7 receptors (Vadodaria et al., 2019a, 2019b). NGN2-induced neurons from monozygotic twins diagnosed with schizophrenia, one clozapine non-responder and one responder, have also been investigated (Kikuchi et al., 2021; Nakazawa et al., 2017). These studies suggest that the phenotype of response or non-response can be depicted in hiPSC-derived cell models at both the level of gene expression and DNA methylation (Kikuchi et al., 2021; Nakazawa et al., 2017) and thus could be used to gain more insight into response mechanisms. More recently, hiPSC-derived neurons from clozapine responding and non-responding individuals with schizophrenia showed differences based on clozapine response in vitro (Hribkova et al., 2022). Moreover, astrocytes generated from the same set of hiPSCs also displayed differences based on clozapine response (Akkouh et al., 2022). These studies further suggest that if treatment response of individual donors is not known or controlled for, it may result in the introduction of additional data heterogeneity, especially when conducting neuropharmacological studies. Taken together, knowledge of the medical history and response to treatment should be taken into account when choosing donors from which to generate/use patient-specific hiPSCs, particularly for neuropharmacological studies.

### 2.4 Consideration of donor sex

**Fig. 1:**
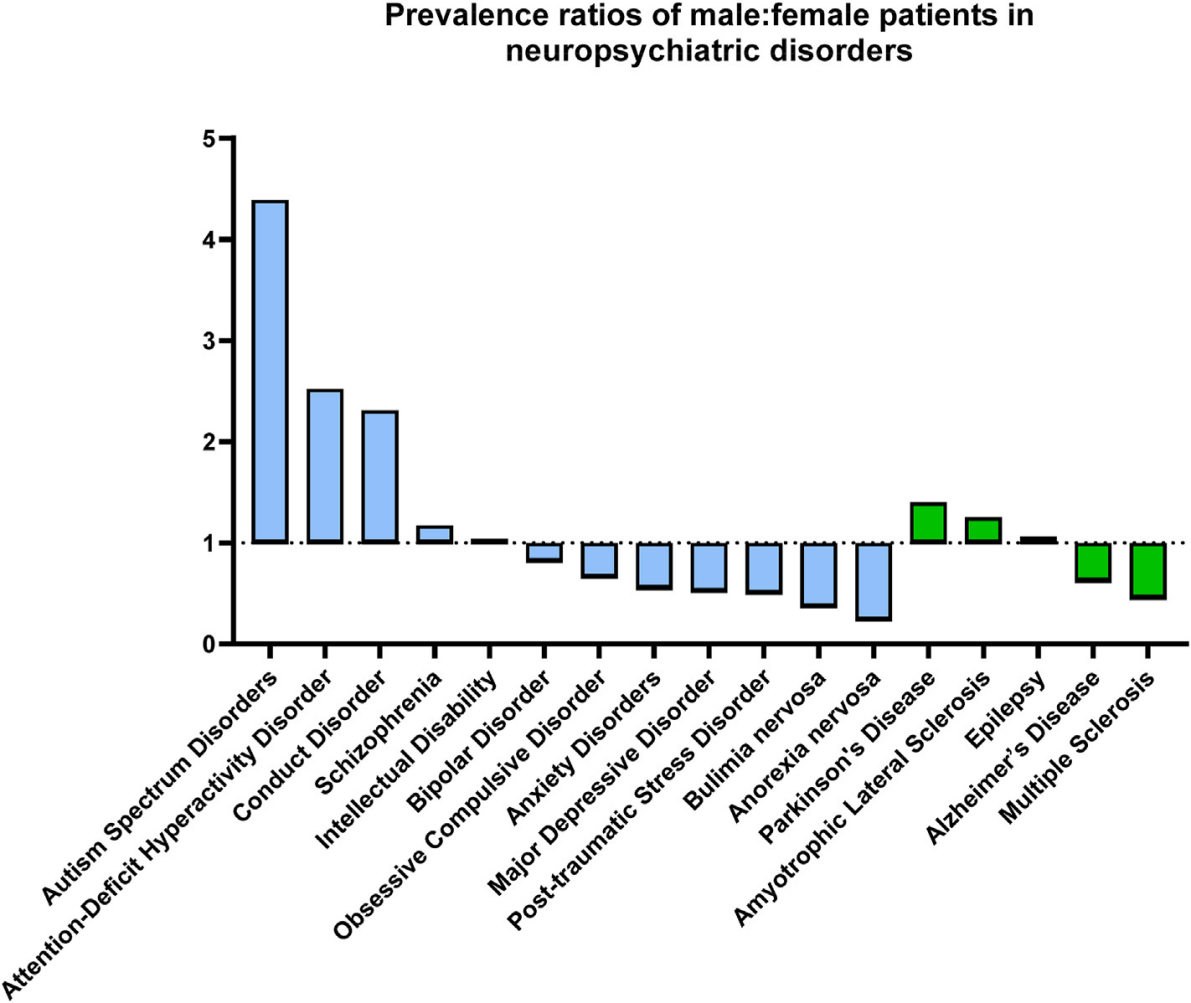
Global prevalence male:female ratios in neuropsychiatric disorders. The graph represents the number of diagnosed male patients for each female in neurological (green) and psychiatric disorders (blue). Male:female ratios of Obsessive Compulsive Disorder, Post-traumatic Stress Disorder and Epilepsy were extracted from (Fawcett et al., 2020; Fiest et al., 2017; Olff, 2017), respectively. Alzheimer's, Parkinson's and Amyotrophic Lateral Sclerosis ratios were reported by the Global Burden Disease (GBD) 2016, while the remaining disorders were updated by the GBD 2019 (https://www.healthdata.org/gbd/2019). (For interpretation of the references to colour in this figure legend, the reader is referred to the Web version of this article.)

Meta-analyses have reported sex differences in the prevalence of multiple NPDs. For example major depressive disorder is predominantly diagnosed in women, and antisocial personality disorder is more frequently diagnosed in men (Albert, 2015). In addition, it has been reported that males are two to four times more likely to develop or be diagnosed with neurodevelopmental disorders such as Autism Spectrum Conditions (ASC) (Loomes et al., 2017; May et al., 2019; Tesic et al., 2019) and ADHD (Liu et al., 2018). Moreover, there is evidence for sex-specific effects of treatment in schizophrenia (Hoekstra et al., 2021). Fig. 1 illustrates the number of male patients diagnosed for every female in several common NPDs. Differences in sex ratios may also vary according to disorder subtypes, age of onset, geographic location, and the survey source; clinic- or community-based (Asadi-Pooya et al., 2012; Biederman et al., 2004; Loomes et al., 2017; May et al., 2019; McLean et al., 2011; Sommer and DeLisi, 2022; Tesic et al., 2019).

Although the origins of sex differences in NPDs are still largely unknown, various theories have been described throughout the last decades based on physiological differences between males and females, especially mechanisms linking genetics to environmental factors (Bale et al., 2010; Christiansen et al., 2022; May et al., 2019; Riecher-Rossler, 2017; Warrier et al., 2022). It is therefore essential to acknowledge differences between males and females at both the clinical level and in diverse molecular/cellular phenotypes, and to plan experiments accordingly to incorporate sex as a biological variable (SABV) (Bale and Epperson, 2017; Galea et al., 2020; Rechlin et al., 2022; Shansky, 2020; Shansky and Murphy, 2021). In all research fields, controlling for biological sex during data analysis is of utmost importance. In hiPSC research, we would suggest the use of hiPSCs from both male and females in a balanced ratio between cohorts, to capture variability that might occur due to sex differences.

In addressing SABV, another important aspect to consider is that of X-chromosome inactivation (XCI). In female somatic cells, one of the X chromosomes is randomly inactivated by the lncRNA *XIST,* resulting in one active (Xa) and one inactive (Xi) chromosome. This phenomenon has been found to withstand reprogramming and remain present in the derived hiPSCs. However, it has been shown that many cultured female hiPSCs show progressive erosion of X chromosome inactivation (Xi erosion; XiE) with resulting reactivation of X chromosomal genes (Geens and Chuva De Sousa Lopes, 2017; Mekhoubad et al., 2012; Wutz, 2012). A comprehensive study analysing more than 700 hiPSC lines revealed that this erosion phenomenon seems to affect mainly hESCs. However, a significant number of hiPSC lines also showed dysregulated XCI (Bar et al., 2019). The XiE phenomenon can have significant consequences for female hiPSC-based cell systems such as upregulated X-linked oncogenes, accelerated proliferation, and an impaired differentiation potential (Anguera et al., 2012; Salomonis et al., 2016). Moreover, the erosion of XCI can also obscure in vitro phenotypes of X-linked diseases (such as Lesch-Nyhan syndrome) when modelled with female hiPSC lines (Mekhoubad et al., 2012). We thus propose that female hiPSC models should use early passage clones and include a thorough analysis of XCI, ideally in a routine manner. While classical methods such as *XIST* FISH or real-time PCR can give some limited insights into the XCI status, a more comprehensive strategy would be analysis of RNA-seq data. An RNA-seq analysis platform has recently been developed that allows the quantitative assessment of *XIST* RNA expression levels, X chromosomal allelic expression and dosage compensation by comparing the expression of X-linked genes to male control lines (Bar et al., 2019). Such analyses, along with high-resolution genetic analyses such as SNP-based virtual karyotyping, should greatly enhance the quality control of female hiPSCs and thus contribute to the standardisation of hiPSC-based in vitro models.

### 2.5 Considerations when working with individuals with rare CNVs

Copy number variants (CNVs) are sub-microscopic structural variants, i.e. genomic deletions, insertions, or duplications, larger than 1 ​kb in size resulting in a deviation of the copy number of the affected segment (Feuk et al., 2006). Rare variants occur in less than 1% of the general population and large variants are usually defined by a size larger than 100 ​kb (Marshall et al., 2017; Szatkiewicz et al., 2014; Vacic et al., 2011). At least 12% of the human genome is potentially variable in copy number. Hence, CNVs contribute significantly to the interindividual genetic diversity and comprise a larger proportion of the genome than all known single nucleotide variants combined (Redon et al., 2006). Depending on gene dosage effects, CNVs can be associated with changes in mRNA and protein expression of directly affected genes, and dosage sensitivity has been proposed to be a determinant of CNV pathogenicity (Rice and McLysaght, 2017). However, CNVs located in non-coding regions can also impact gene expression of adjacent genes by altering the gene dosage of enhancers/silencers or via changes in the three-dimensional chromatin structure, which can alter the accessibility of promoters for regulatory elements (Spielmann et al., 2018).

Multiple GWAS have shown an enrichment of large rare CNVs in several NPDs, including schizophrenia (SZ) (Marshall et al., 2017; Szatkiewicz et al., 2014; Vacic et al., 2011), ASC (Leppa et al., 2016; Sanders et al., 2015; Warrier et al., 2022), ADHD (Williams et al., 2010) and bipolar disorder (Charney et al., 2019). Initial hiPSC-based studies have now reported differences in cellular morphology, gene expression, cell metabolism, synaptic functioning and calcium signalling in hiPSC-derived neurons carrying NPD-associated CNVs, such as the 15q11.2-q13.1 locus (Das et al., 2015; Germain et al., 2014), 16p11.2 locus (Deshpande et al., 2017; Li et al., 2021), 22q11.2 locus (Khan et al., 2020), 1q21.1 locus (Chapman et al., 2022), *CHRFAM7A* gene (Ihnatovych et al., 2019), and *PARK2* gene (Palladino et al., 2020). Therefore, hiPSCs from deletion or duplication carriers of neuropsychiatric risk genes are a valuable resource that can bridge the gap between genetic association and cellular function directly in patient-derived cells.

Before using hiPSCs derived from individuals carrying CNVs, several aspects must be considered, including genotyping and experimental design. Firstly, there should be strong evidence for CNV-disease association, either from GWAS or well-powered case-control studies. Even though the relative risk conferred by CNVs in coding regions is thought to be higher than the impact of functional SNP variants, it should still be considered that disease risk for common neuropsychiatric disorders is modified by a heterogeneous and complex mixture of both genetic and environmental variation. Therefore, it is recommendable to focus on gene variants which show robust disease association and exhibit high biological evidence for an involvement in general neurodevelopmental processes, such as axonal outgrowth/pruning, synaptogenesis and myelination (Silbereis et al., 2016), or which are involved in cellular processes necessary for neurotransmission, such as glucose metabolism (Bak et al., 2006) or calcium signalling (Mattson et al., 2000). Secondly, a discovery sample should be screened for the variant of interest, which can be achieved either by PCR-based copy number assays, or by a genome-wide chip array whose resolution must be chosen according to the size of the CNV, in order to reliably capture the variant. Whereas the former approach might be more cost-efficient and faster when a large cohort must be screened, the latter brings the benefit of a whole genome readout not only for CNVs but also for SNP variants, allowing the researcher to statistically control for genetic background in subsequent analyses.

Thirdly, in cases of extremely rare CNVs, it can be advantageous to generate hiPSCs from carriers and non-carriers of the specific CNV within the same family to control for at least part of the genetic background (see discussion above on selection of appropriate control hiPSC lines). If there is uncertainty about a potential gene-dosage effect of the CNV of interest, one approach could be to assess the expression pattern when choosing the cells of origin for the reprogramming procedure. This way gene expression can first be assessed in the peripheral cell model (e.g. in dermal fibroblasts or leukocytes). However, this pattern may be impacted by both cell type-specific mechanisms as well as epigenetic factors. Finally, when hiPSCs have been derived from CNV carriers and non-carriers, it is mandatory to re-check and confirm the existence of the CNV in these cell lines. As chromosomal aberrations occur frequently in hiPSCs (Halliwell et al., 2020; Mayshar et al., 2010), the karyotype of all cell lines under investigation should be checked every 10 passages or whenever a cell line shows unexpected alterations in morphology, growth, or expression patterns.

### 2.6 Handling of patient information, consent and GDPR

hiPSCs are associated with the genetic and clinical phenotype of the donor. Furthermore, additional detailed phenotypic, clinical, and behavioural data, including family history, age of disease onset, medications, and diagnostic results may be collected (Isasi et al., 2014). The donors are ‘natural or legal persons’ and the data are ‘*personal’ and ‘health’ data*, which are considered especially sensitive and require legal protection. The rule is that all personal data are regulated by data protection law, meaning donors are entitled to a high degree of privacy protection and to security of the data associated with the human cell line. In this section we will briefly summarise the rules and regulations of hiPSC use in Europe (authors' location).

In Europe, the generation, storage, and handling of hiPSCs are subject to data protection that is regulated by the European General Data Protection Regulation (*GDPR*; EUR-Lex 3216R0679, Directive 95/46/EC) (Poullet, 2006). The directive came into effect in 2018 and it allows European Member States to implement data protection requirements into national law with differences between jurisdictions. Data samples must be traceable to the sample donor (Morrison et al., 2016) and data associated with hiPSC processing, including collection, storage, editing, analysing or otherwise working with personal data must be designed and structured with consideration of these directives (Box 2) and include safeguards to protect data using pseudonymization or full anonymization where appropriate.

Box 2 **- EU regulation of the generation, storage, and handling of hiPSCs**

Council Regulation (EC) 2016/679 of 27 April 2016 on the protection of natural persons with regard to the processing of personal data and on the free movement of such data, and repealing Directive 95/46/EC, OJ L119/1.

Council Directive (EC) 2004/23 of 31 March 2014 on setting standards of quality and safety for the donation, procurement, testing, processing, preservation, storage and distribution of human tissues and cells, OJ L102/48).

Council Directive (EC) 2006/86 24 October 2006 implementing Directive 2004/23/EC of the European Parliament and of the Council as regards traceability requirements, notification of serious adverse reactions and events and certain requirements for the coding, processing, preservation, storage and distribution of human tissues and cells, OJ L294/32).

Alt-text: Box 2

*Pseudonymization* means that the data can still be used to identify individuals and it is possible to combine data that exist in different records. However, such information is still considered personal and thus the processing of such data is subject to “data protection” regulation. Encoded data are connected to a specific individual with a code key that allows the holder of the code key to decode the records and identify each data subject. False names can also be used to protect personal data. European regulations on the traceability of biological material indicate that, for quality and safety purposes, all generated hiPSCs can be pseudonymized, which is recommended practice in the EU. This is consistent with WHO Guidelines on traceability of human organs, tissues and cells (World Health, 2010). The GDPR promotes the principles of accountability and transparency that require effective governance and record keeping by researchers. Data being used for research are stipulated to be stored ’for as long as necessary’. The GDPR also includes the principle of data minimisation meaning that personal data shall be adequate, relevant and limited to what is necessary in relation to the purposes for which they are processed.

Biological samples and data are collected with the donor’s consent for medical research and product development for healthcare and health benefiting purposes in Biobanks. When processing of personal data is based on consent the donor has the right to revoke it at any time. Consent must be obtained before information about a data subject can be collected and processed. For donors of tissue that will be used to generate hiPSCs, consent must be informed and explicit for donation of the biological sample for reprogramming and for the collection and processing of personal data and genetic material that will be used to characterise the cell line. An individual participant, or a proxy on behalf of a person who is not able to give valid consent (e.g. a minor or a person with a developmental disability or dementia), are requested to sign/initial the consent form that should contain a separate statement that agrees sample collection, reprogramming, storage, and association with the personal and/or medical data of the participant. The consent can be broad, but it must meet the criterion to be explicit about what is being consented to. Therefore, explicit consent provisions to allow hiPSC lines and associated data to be made available to the wider scientific community (including, if allowed, commercial partners) should be added. Since genetic and biometric data can be ’inherently identifying’, explicit consent for their collection and processing is recommended.

Lastly, permission to share cell lines and data with researchers in other countries, the private sector and post-study deposition of the lines and data in a biobank is appropriate and highly recommendable. Several hiPSC Biobanks currently exist, such as the European Bank for induced pluripotent Stem Cells (EBiSC; https://ebisc.org/), which aims to increase collaboration and promote harmonisation of hiPSC research standards, including handling of personal data (Steeg et al., 2021). A further function of biobank governance is the compliance with data protection law and simultaneously managing operational, legal, and frequent ethical matters.

## 3 Best practices for hiPSC-based experimental design

PSCs present a unique and complex set of challenges for experimental standardisation. They are heterogeneous in nature, with variability arising from both genetics and cell culture history (Anderson et al., 2021; Volpato and Webber, 2020). For hiPSCs specifically, variability may arise from tissue of origin and completeness of epigenetic wiping during reprogramming, as well as genetic background. Epigenetic mechanisms play a crucial role modulating disease-associated factors and pathways. Alterations of the molecular epigenetic machinery and regulatory function are associated with the pathophysiology of neuropsychiatric disorders (Qureshi IA & Mehler, 2018; Zhu et al., 2020). hiPSC reprogramming is recognized as a major epigenetic remodelling process that is necessary to adjust the epigenetic state of the parental cell to a state compatible with pluripotency (Gao et al., 2017). However, it has been shown that some epigenetic marks in hiPSCs differ from those in hESCs, suggesting the presence of residual somatic epigenetic signatures. This epigenetic memory can limit the cells reprogramming efficiency and potency. Particularly, DNA methylation is considered a crucial epigenetic barrier associated with low efficiency in cell reprogramming (O'Malley et al., 2013; Brix et al., 2015; Gomes et al., 2017). It has been suggested that epigenetic memory predispose the cells to differentiate towards the lineage of the cell of origin. However, late-passage cells show minimal bias to their differentiation potential, demonstrating that epigenetic memory is attenuated over multiple passages (de Boni et al., 2018; Efrat, 2020; Poetsch et al., 2022). Furthermore, genome-wide analyses with hiPSCs derived from different somatic tissues from multiple individuals have shown that the major driver of variation among lines is the donor's genetic background, while the epigenetic memory or intrinsic variability of the hiPSC system have minor contributions (Gao et al., 2017; Scesa et al., 2021).

The corresponding ‘biological age’ of PSC-derived models should also be taken into account when planning experiments; for example, hiPSC-derived neurons typical reflect an early, prenatal developmental stage (Steg et al., 2021), and can therefore be used for investigating NPDs with a neurodevelopmental origin, whereas directly induced neurons typically represent more mature neurons (Mertens et al., 2016) and are therefore more suitable for investigating age-related disorders (for review see Flitsch and Brüstle, 2019). Further layers of complexity arise from stochastic variation between biological replicates (e.g. Stumpf et al., 2017) and the tendency to accumulate mutations that confer growth advantages (Halliwell et al., 2020; Jerber et al., 2021; Kuijk et al., 2020). Recent studies have highlighted that the accumulation of mutations in mitochondrial, as well as nucleic DNA may contribute to significant transcriptomic differences between cells, both within and between hiPSC clones (Wei et al., 2021; Perales-Clemente et al., 2016; Carelli et al., 2022). Mitochondrial SNVs accumulate during mitochondrial DNA replication throughout life and are thus heteroplasmic in nature, present only a proportion of total mitochondrial DNA (Stewart and Chinnery, 2021). However dramatic changes in mitochondrial heteroplasmy have been observed during reprogramming of somatic cells to hiPSCs (Wei et al., 2021, Perales-Clemente et al., 2016), further contributing to hiPSC heterogeneity. As such mitochondrial as well as nucleic DNA should be considered during quality control of hiPSC lines.

These considerations, along with increasing calls for assurance of scientific reproducibility, make a clear case for the incorporation of QC measures into experimental design of any hiPSC experiment. Means to assure core attributes of PSCs such as viability, sterility, and pluripotency have been explored elsewhere (for example see (Abranches et al., 2020; Anderson et al., 2021; O'Shea et al., 2020; Pamies et al., 2018; Steeg et al., 2021; Yaffe et al., 2016)). In this section we explore considerations for design of hiPSC experiments for neuropsychopharmacology and neurodevelopmental modelling.

### 3.1 Improving data reproducibility and robustness

The novelty and inherently heterogeneous nature of hiPSC technology has complicated basic experimental planning, and there is currently a lack of consensus regarding the minimum standard required for robust hiPSC study design, particularly regarding factors such as the minimum number of biological replicates. Current practice is to use multiple clones from the same donor, which is also entrenched into the guidelines of several journals (e.g. Stem Cell Reports) (McNeill et al., 2020). However, the inclusion of more than one clone per donor in hiPSC transcriptomic datasets has been shown to result in a significant increase in the detection of spurious differentially expressed genes, suggesting that the hiPSC field may currently be reporting a high number of false positive results (Germain and Testa, 2017). Indeed, hiPSC clones of a single individual created from different somatic cell types, such as blood or skin, are more similar in their expression profile than hiPSC-lines created from identical somatic cell types from different donors (Rouhani et al., 2014). In order to counteract this, and increase the reproducibility of hiPSC research, a consensus must be reached by the community on how many replicates (clones and biological donors) are required to produce robust results.

In the study by Germain and Testa (2017), which first identified the problems of using more than one clone per donor, they further investigated how many donors would be required for adequate sensitivity if only one clone per donor was used (Germain and Testa, 2017). The results revealed that when comparing single clones from unrelated individual donors, sensitivity appeared to largely plateau after six individuals per group. The inclusion of two or more clones per individual incrementally increased sensitivity, however, at a large cost to specificity. A robust experimental design should attempt to balance the trade-off between specificity and sensitivity, and therefore the emphasis should be on the inclusion of more individual donors per group, instead of the number of clones per individual. The authors of the study concluded that for using single clones from unrelated individuals, a minimum of four donors per group should be utilised (Germain and Testa, 2017).

Although a minimum of four donors and single clones may be sufficient for detecting the biological consequences of genetic variants with large effect sizes, for example in monogenic disease, further studies have suggested that increased numbers of individuals will be required for the study of common genetic variants such as SNPs. In 2018, Schwartzentruber et al*.* conducted the first large scale study of common genetic variants in a hiPSC-derived neuronal cell type, sequencing 177 hiPSC-derived sensory neurons (Schwartzentruber et al., 2018). They reported a large degree of resultant culture heterogeneity due to variable differentiation capacity of the hiPSC cell cultures, thereby inhibiting the power of such studies to capture the biological effects of common genetic variants. The authors concluded that recall-by-genotype hiPSC studies will require relatively large sample sizes and suggested that between 20 and 80 individual donors should be used (Schwartzentruber et al., 2018). However, the labour and cost associated with such high numbers of independent donor lines make experiments of this scale unfeasible for most research groups. These findings may highlight a need to widen access to larger numbers of hiPSC lines, for example through centralised resources such as the previously mentioned Biobanks/stem cell repositories. An alternative approach has recently been used while studying 22q11.2 deletion syndrome (Nehme et al., 2022). In this study, the authors performed a pilot experiment using 2 hiPSC lines from either carriers of the 22q11.2 chromosomal deletion, or non-carriers. Based on RNA-sequencing data generated from all 4 lines at 3 distinct time points of differentiation, the authors performed a power calculation to determine that a sample size of >20 control and donor lines were needed in order to detect transcriptomic changes (Nehme et al., 2022). This approach highlights how a data driven approach could be utilised in order to provide insight into what would be an appropriately power study for a given phenotype or assay.

Even individual hiPSC lines derived from the same individual can differ greatly, for example in their differentiation ability, which is thought to be due to subtle alterations introduced during reprogramming and/or as yet unknown epigenetic differences (Jerber et al., 2021; Liang and Zhang, 2013). Indeed, even the same hiPSC line seeded into different wells can demonstrate varying well-to-well capacity to differentiate into the desired cell type (Chan and Teo, 2020). Other sources of hiPSC and hiPSC-derived cell heterogeneity include technical parameters such as cell culture medium (Schwartzentruber et al., 2018), passage number (Volpato and Webber, 2020), weekend feeding, and use of frozen neural progenitor cells (Volpato et al., 2018). As hiPSC generation and differentiation are multi-step processes, small variations can be introduced at many stages and accumulate (Popp et al., 2018), possibly resulting in cultures that are different due to technical aspects and not diagnosis/genetics. One source of variability could be from batch-to-batch variability – where each batch is defined by a separate differentiation of hiPSCs. In this case, we suggest differentiating patient and control lines within the same batch. Nevertheless, a large degree of data variability can be expected in hiPSC research; however, robust experimental design and optimised statistical methods can help remove the technical noise inherent to hiPSC experiments.

At least two statistical methods have been introduced to try and control for hiPSC data heterogeneity. Firstly, Germain and Testa (2017) used a statistical approach called limma's *duplicateCorrelation*, in order to determine whether more than one clone per donor could be used whilst still controlling for false positives (Germain and Testa, 2017). This is a mixed-models approach which treats the individual as a random-effect variable. The approach was found to reduce the false discovery rate whilst offering increased sensitivity, leading the authors to recommend the use of this model (with a more stringent threshold) when analysing datasets including more than one clone per donor. However, it should be noted that the use of only one clone per donor still showed superior specificity. Secondly, Volpato et al. (2018) attempted to control for noise using a factor-based analysis method called remove unwanted variation (RUV) (Volpato et al., 2018), which aims to remove technical variation whilst retaining variation associated with a biological covariate of interest (Risso et al., 2014). When applied to transcriptomic and proteomic datasets, two individual hiPSC lines could be separated, whereas previously they could not. Principal component analysis (PCA) revealed that the individual laboratories where the hiPSC lines had been generated were originally identified as a major cause of variation. As highlighted above, Volpato and Webber (2020) called for the use of common, “Rosetta-stone” hiPSC control lines as an approach to reduce variability between laboratories (Volpato and Webber, 2020). Post-RUV PCA for each laboratory showed vast improvements in ability to segregate the data by both time point and genotype, suggesting that RUV was able to remove the nuisance technical noise caused by individual laboratories, thereby revealing biological signals.

In conclusion, the inherently variable nature of hiPSC research requires careful planning of experiments and clearer recommendations on the minimum number of replicates required to ensure reproducibility of results. From the current evidence, we suggest that emphasis should be on an increased number of individual donors rather than the number of clones from each individual. We further recommend that only one clone per donor be utilised, particularly for -omics approaches, unless adequate statistical methods (such as limma's *duplicateCorrelation*) are used to address data variability and interdependence of clones. For the study of monogenic diseases, at least three individual donors per group should be used. For common genetic variants such as SNPs, as many individual donors as possible should be included; however, we acknowledge that feasibility is a problem, therefore we recommended a minimum of four donors per genotype for preliminary experiments. We further highlight the need for careful selection of control lines, with a particular emphasis on ensuring that the genetic background of the control hiPSC lines are appropriate for the experimental hypothesis being tested. For hiPSC experiments involving genome-edited isogenic lines, Chan and Teo (2020) suggested that when studying gene function, two CRISPR-edited isogenic knock-out hiPSC lines combined with one sham isogenic control (exposed to CRISPR constructs but not edited) may suffice (Chan and Teo, 2020). However, it should be noted that it is possible that different isogenic hiPSC lines may still vary greatly in their ability to differentiate, therefore the optimum number of CRISPR-edited hiPSC lines per donor remains uncertain. We therefore recommend that at least two donors be used, from which three lines are derived: parental hiPSCs, edited hiPSCs, and an unedited sister control. We further like to underline the importance of validating edited hiPSCs, for the targeted protein/gene of interest and known/predicted off-target effects before they are used in experiments and the standardisation of these procedures. Publishing edited hiPSCs as laboratory resources and in stem cell repositories could largely contribute to the overall quality of edited hiPSCs and ensure that they follow internationally recognized standards. Lastly, with regards to controlling noise caused by technical variance, we recommend that large datasets are adjusted using RUV to help identify biological signals. For a summary of recommendations regarding minimum number of replicates and use of appropriate statistical methods, please see Table 1 and Table 2 respectively.

**Table 1:** Recommended minimum of replicates for hiPSC experiments.

Type of Study	Recommended Minimum Number of Replicates (Clones and Donors)
Monogenic disease	1 clone per donor; 3 donors
Polygenic disease (e.g. SNPs)	1 clone per donor; ≥4 donors
CRISPR genome-edited	3 CRISPR-edited lines per donor (unedited, edited and sham control); 2 donors

**Table 2:** Recommended statistical approaches for hiPSC data analysis.

Statistical Approach	Function	Use
limma's *duplicateCorrelation*	Accounts for interdependence of clones, allows multiple clones per donor to be used for increased sensitivity whilst minimising false positive results	When more than one clone per donor is used
Remove Unwanted Variation (RUV)	Removes technical variation whilst retaining variation associated with a biological covariate of interest	Large datasets

## 4 Best practice when performing neuropharmacological studies relevant for neuropsychiatric disorders

**Fig. 2:**
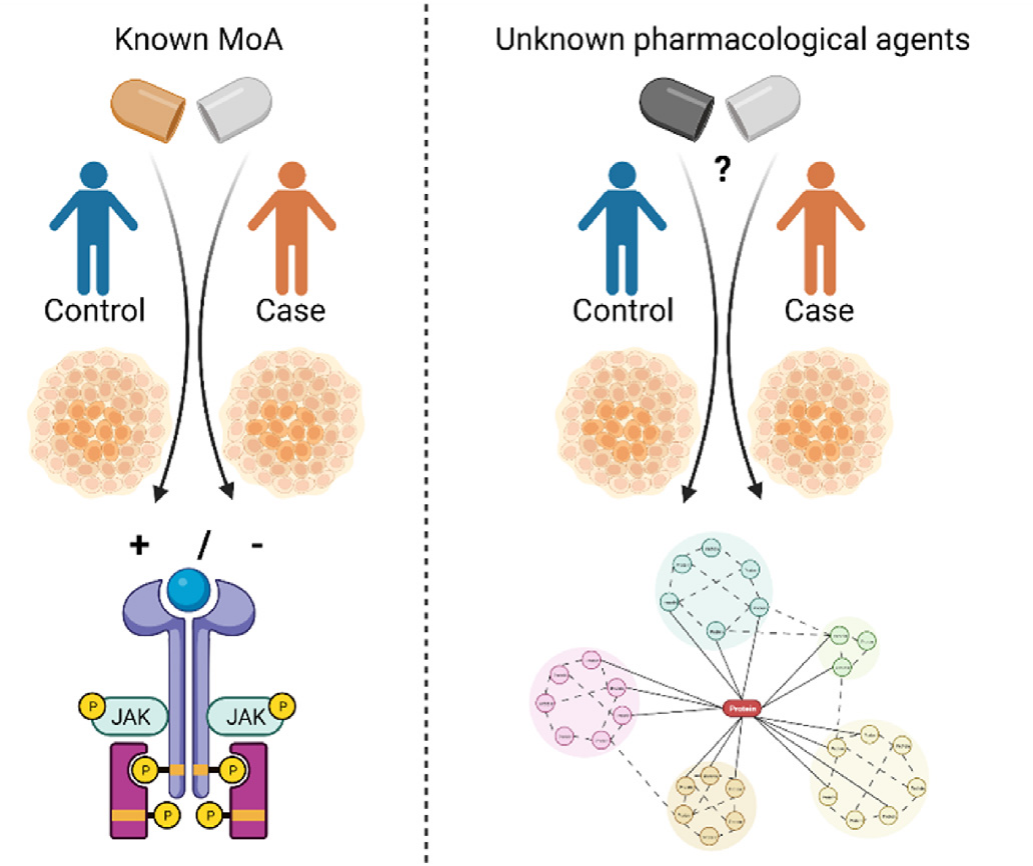
The two main classes of neuropharmacological study approaches. Abbreviation: MoA, mechanism of action.

hiPSC-derived neural cells – we used this term to encompass hiPSC-derived neurons (excitatory and inhibitory) and glial cells (astrocytes, microglia and oligodendrocytes), grown either in 2D or 3D – offer a translationally relevant model for assessing the effects of pharmacological agents. Schematically, most neuropharmacological studies can be classified into two main groups, according to the goal of the study (Fig. 2). In the first case, the study is aimed at characterising a given molecular phenotype of hiPSC-derived neural cells from healthy donors and/or from patients with NPDs, and the pharmacological agents used are selected according to their well-known mechanism of action (MoA) to trigger specific molecular pathways. In the second case, the study is aimed at characterising novel unknown pharmacological agents or repurposed drugs, with the hiPSC-derived neural cells used as a translational substrate according to their relevance for the NPD under assessment.

Canonical pharmacological tests should be chosen within the context of translational modelling principles, previously applied to preclinical animal models (Geyer and Markou, 1995). Most of these tests (e.g., dose-response curve, time-dependent curve, inhibition-response studies etc.) are essential to produce the convergent set of information necessary to deliver a convincing and reliable outcome, as described in detail later. In fact, if only a subset of these tests were run, the overall inferential values of the study would be of limited value, in the worst case leading to questionable conclusions. To avoid this risk, the discussion of the results should include a gap analysis regarding the missing information, sharing the awareness that the data may constitute a suggestive but preliminary assessment of pharmacological effects, rather than robust and definitive results.

Of note, the majority published studies applying hiPSC technology in patient-specific models of neuropsychiatric disorders tend to focus on neuronal phenotypes (Gonzalez et al. 2017). While beyond the scope of these guidelines, it is important to highlight the emerging contributions of non-neuronal cells in the pathology of neuropsychiatric disorders, including microglia (for review see: Mondelli et al. 2017; Hanger et al. 2020). Several protocols have now been developed to derive microglia like cells from hiPSCs (Abud et al., 2017; Haenseler et al., 2017; Muffat et al., 2016) (for review see Hasselmann and Blurton-Jones, 2020), including developmentally informed methods for large-scale production of cryopreservable hiPSC-derived microglia (Mathews et al., 2023). As an example of the utility of these cells, Göttert et al. (2022) investigated the effects of lithium treatment on the form and function of human primary microglia, hiPSC-derived microglia, and an immortalised microglia cell line. The data suggest that lithium treatment counteracted interferon-γ (IFN-γ) mediated up-regulation of indoleamine-2,3-dioxygenase (IDO1) expression and activity across the aforementioned microglia culture models, highlighting that treatment effects of lithium may include shifting microglia back towards a homeostatic functional state (Göttert et al., 2022). Microglia derived from hiPSC may also be used in conjunction with high-throughput CRIPSR interference and activation screens, which has the potential to identify novel therapeutic targets (Dräger et al., 2022). Although we highlight examples related to microglia above, we note that similar importance should be attached to the study of other non-neuronal cells which may be derived from hiPSC including astrocytes (Akkouh et al. 2022) and oligodendrocytes (McPhie et al., 2018). As such whilst we explicitly refer to neuronal cells in the following paragraphs, it should be assumed that these guidelines apply to both neurons and glial cells in culture.

### 4.1 Probing the neurobiology of hiPSC-derived neurons using pharmacological agents with a known MoA

The starting assumption for these types of neuropharmacological studies is that a given pharmacological agent well-known for its selective MoA can be used as a tool to probe the presence of a given molecular mechanism in hiPSC-derived neural cells, the phenotypes of which were not previously fully characterised. Selective agonists and antagonists can be used, generally in the low micromolar range, to drive molecular mechanisms that could modify the observed sub-cellular phenotypes or functions. The selection of the pharmacological tools could be determined from prior knowledge of genetic or pathological mechanisms underpinning the NPD. For example, several interesting studies have been conducted using hiPSC-derived forebrain neurons from donors with diagnosis of a familial form of psychosis that carry mutations in the disrupted-in-schizophrenia-1 (*DISC1*) gene (Kim et al., 2021; Wen et al., 2014). In the initial study, reductions in synaptic number and synaptic vesicle release were observed, which could be reverted by correction of the *DISC1* mutation via gene editing (Wen et al., 2014). In the follow up study using the same hiPSC-derived forebrain neurons, significantly increased phosphodiesterase 4 (*PDE4*) transcript expression was found, possibly mediating the observed reduction in synaptic density. The clinically effective PDE4 inhibitor rolipram (prescribed for Chronic Obstructive Pulmonary Disorder) was used as a tool to inhibit PDE4 enzymatic activity in vitro at 100 ​μM, resulting in the functional rescue of the observed synaptic deficits (Kim et al., 2021). The in vivo relevance of these effects was confirmed in knock-in mice carrying the human *DISC1* mutation, supporting a critical role for cAMP-dependent pathways in *DISC1*-related psychosis.

As the selected pharmacological agent is also usually clinically effective, it is tempting to link the observed in vitro result with clinically relevant observations, suggesting a causal relationship. However, this is not always the case, as the human CNS is a highly complex system and therefore the translational relevance can be difficult to establish. For example, in one of the first in vitro experiments on hiPSC-derived cortical neurons from donors with schizophrenia, an abnormal phenotype with shorter dendrites and less synaptic spines was observed (Brennand et al., 2011), mimicking the defective connectivity described post-mortem in the cortex of patients with SZ. This phenotype could be reversed by in vitro exposure to a single high dose concentration of loxapine, an antipsychotic drug, suggesting a possible link between the in vitro response and the clinical effects. However, this in vitro effect could not be replicated using other clinically effective antipsychotics (Brennand et al., 2011). In addition, loxapine has not been shown to improve negative and cognitive symptoms at the clinical level, which are the main symptoms that are believed to be associated with the reduced cortical connectivity observed in schizophrenia (Collo et al., 2020; Fenton et al., 2000). Therefore, simple analogies with good face validity may not always work in translation, requiring a more complex neuropharmacological construct that would include converging information aimed to bridge preclinical exposure data with pharmacokinetic and clinical data in humans.

### 4.2 Profiling novel pharmacological agents or repurposed drugs with an unknown MoA using hiPSC-derived neurons

Initial experiments should aim to characterise specific cellular and molecular responses (e.g. increased synaptic density or specific protein phosphorylation) to gold standard drugs known for their therapeutic clinical effects on the disorder of interest. This will provide a reference benchmark for the building of the translational model. After the development of this benchmark, the compound(s) of interest should be tested at a wide range of concentrations, assessing the same specific cellular and molecular parameters that were used to profile the response to gold standard drugs. It is expected that phenotypic or molecular changes caused by the pharmacological agent of interest should partially overlap changes caused by the gold standard drugs, in order to propose a translational relevance. This paradigm is increasingly used both in academic and industrial drug discovery (Farkhondeh et al., 2019).

In an example of this paradigm, hiPSC-derived mesencephalic dopaminergic (DAergic) neurons can be obtained with high reliability from healthy donors and from patients with CNS disorders. They possess a well characterised A9 mesencephalic phenotype which has been extensively described in the literature (Kriks et al., 2011; Soliman et al., 2017). In a series of experiments, structural plasticity of dendritic arborisation in these DAergic neurons were profiled in response to DAergic agonists such as pramipexole and ropinirole, drugs clinically used in Parkinson's disorder and to potentiate the antidepressant response to SSRI antidepressants in patients partially responding to the therapy. Changes of dendrite length and number of DAergic neurons were selected as structural plasticity experimental endpoints, since impairment of neural plasticity is a recognized key cellular mechanism produced by chronic stress and observed in Major Depression Disorder (MDD). A significant increase in dendrite length and number was observed with ketamine, a drug clinically effective in treatment resistant depression (TRD), indicating an improvement in neural plasticity (Cavalleri et al., 2018). This paradigm was used to study the MoA of the ketamine metabolite (6R-2R)-hydoxynorketamine (HNK), an agent considered as a putative antidepressant, but with an unknown MoA. The results obtained from several experiments in hiPSC-derived DAergic neurons indicated a critical role of the BDNF and mTOR pathways (Cavalleri et al., 2018; Collo et al., 2018). These effects were observed in vitro at doses estimated to be in the same range as the clinically effective concentration, further suggesting a potential translational value. Moreover, this study provided information regarding the possible effective therapeutic dose to be used in the human studies with HNK. These studies, together with other preclinical data, supported the rationale for the current clinical development of HNK (https://clinicaltrials.gov/ct2/show/NCT04711005).

It is however important to note that even though phenotypic and molecular changes by a compound of interest can overlap with gold standard drugs, this is not necessarily the case for their exact pharmacodynamics and pharmacokinetics. These characteristics can greatly affect the safety, efficacy and thereby also the translational relevance of the compound of interest.

### 4.3 Building evidence of translational value using standard neuropharmacological tests on hiPSC-derived neurons

A good practice neuropharmacological paradigm includes a set of tests aimed to explore the relationship between any pharmacological agent and the neurobiological substrates considered translationally relevant for its expected therapeutic effect. The paradigm is built on the basic textbook principles of pharmacodynamics and pharmacokinetics, as well as on the understanding of the neurobiological substrate selected to mimic (model) a critical trait of the NPD under study. Below we have proposed a list of key steps recommended for achieving a satisfactory outcome for a publication (Table 3).

**Table 3:** Recommended steps for neuropharmacology studies using hiPSCs. ED_50_ ​= ​effective dose 50, the median effective dose that produces a therapeutic effect in 50% population; IC_50_ ​= ​inhibitory concentration 50, the concentration of the drug needed to inhibit a biological process by 50%.

(1) Identify and select from the literature relevant correlates/analogies between the cellular pathological events occurring in the NPD of interest (in vivo) and those observed in the hiPSC-derived cells (in vitro). The hiPSC-derived cell type(s) used should be specifically selected to represent the brain circuit involved in the disorder. To this aim, supporting evidence should be collected from human neuroimaging, animal models, biomarker and post-mortem studies, in order to provide construct validity for the translational model.
(2) Experimentally validate the presence of the relevant target in the hiPSC-derived cell model and demonstrate dose-dependent and time-dependent effects of the pharmacological agents on biological markers that characterise specific aspects of the relevant, hiPSC-derived cell phenotype. It is important that the carefully validated biological markers of the hiPSC-derived cell phenotype match the MoA of the pharmacological agents under evaluation. This is a critical piece of evidence necessary to map the dose-effect relationship between the pharmacological agent and the biological substrate, generally expressed with an ED_50_/IC_50_. If possible, use two different pharmacological agents with the same MoA to show generalisation.
(3) Experimentally identify the specificity of the response to the pharmacological agent using inhibitors of the receptor (e.g. antagonists) or of the intracellular pathways (e.g. phosphorylation inhibitors) that are thought to be involved. If the utilised inhibitors have already been characterised in vitro and the IC_50_ determined, a single saturating dose should be used in the current study. If no literature is available, the inhibitor should be used at various concentrations, producing a dose-inhibition curve and determination of the IC_50_, while using a single dose of the pharmacological agents of interest at their ED_50_. To confirm generalisation, it is suggested to use at least two different blocking/deactivating agents or procedures (including knock-out).
(4) Experimentally assess if the acute response in the hiPSC-derived neurons to the pharmacological agent is maintained during repeated dosing. In case of reduction or disappearance of effects over time, tolerance is produced. This should trigger a search for its molecular underpinnings. Moreover, it may have a potential translational effect. If more than one drug with the same MoA was used and tolerance was consistently observed, it is unlikely that this mechanism is involved in the sustained therapeutic effects observed and required clinically.
(5) Collect pharmacokinetic & pharmacodynamic data related to the pharmacological agent of interest from human studies in healthy volunteers and in patients diagnosed with the NPD being studied. This will allow the researcher to identify the estimated brain concentration of the drug that is associated with clinical effect, generally under chronic dosing regimens. These concentration values should be in the range of the active concentrations of the dose-response curve identified in vitro on hiPSC-derived neurons and included in a PK-PD model using the dose-effect relationships observed in vitro with the same relationship reported in clinical studies.

Overall, Table 3 indicates a good practice approach when there are pharmacological agents used as standard-of-care. However, when there is no such treatment identified (for example for some rare disorders), no benchmark can be provided. In this case, ‘well-known’ pharmacological agents with a highly selective profile for a given receptor/pathway could be used as a tool to probe the integrity of certain mechanisms in the hiPSC-derived neurons from donors NPDs, to better characterise the defective phenotype in comparison to those from healthy controls. In this case, steps 1–4 are still valid and recommended. It is important to note that the list of recommended steps we have provided indicate a paradigm commonly used in pharmacological studies, but should not be seen as exhaustive.

### 4.4 Considerations for choosing appropriate cellular readouts relevant for the pathophysiology of NPDs

A key to the successful application of hiPSC-based research is the careful selection of the most appropriate cell-based assays and readouts for the biological question under investigation. In this section, we consider selection of appropriate assays and their experimental outputs when designing hiPSC-based NPD studies.

To realise the full potential of hiPSC-based assays, initial assay selection needs to fit the required experimental outcomes. An important factor is whether an assay is meant to provide insight into biological or disease mechanisms – indeed, the biological mechanism underlying the disorder may exert its primary effects prior to when it is possible or practical to administer a drug candidate – or whether it will be used for drug development. In other words, is a desired drug candidate preventive or corrective? In the first case, biological meaningful readouts that reflect physiological or pathological parameters (e.g. growth or degeneration of axons) are needed. In the second situation, more generic phenotypes (e.g. the size of an organoid) or cellular phenotype may suffice as long as they reflect the underlying pathomechanism and meet other requirements for drug discovery (e.g. ability to be converted into high throughput screening formats).

Assay selection also needs to consider different data types, data volume, and the extent of sample variation expected, determining statistical power. All of these factors will determine the analytical resolution that can be achieved. A good example of this is when performing transcriptional profiling using RNA-sequencing to assess hiPSC neural differentiation. The specific design, technological implementation, and amount of data (expressed as read-depth) needs to be appropriate for the outcome. Gene discovery experiments, especially for Differentially Expressed Gene (DEG) analysis, require high read-depth and numbers of samples and replicates, especially if carried out at the single cell level. In contrast, profiling of cell type distributions and developmental timing is tolerant of lower read-depth and sample numbers, and variation can be overcome by pooling genes into expression modules or selection of a subset of cell type-specific highly expressed genes. Alternative approaches include the sorting of cells into cell populations followed by RNA-sequencing. These same considerations exist across most quantitative data that can be obtained from differentiated hiPSC cultures, including proteomics, cell morphometrics and electrophysiological function. For these reasons, assay design needs to start with a clear understanding of the expected and desired experimental outcome, its sensitivity and specificity, and the suitability of the techniques deployed to collect quantitative data at the required resolution.

### 4.5 Considerations of acute vs chronic/repeated treatment schedules for in vitro studies

Psychopharmacological treatment in vivo is almost always administered chronically, except for the effects of some sedative and anaesthetic medications. Clinical concepts of response, partial response, or non-response to antipsychotics, antidepressants or mood-stabilisers are also predominantly based on chronic treatment. This represents a major hurdle for hiPSC-derived in vitro pharmacological experiments, as acute effects are easier to model, whereas chronic treatment is usually laborious and difficult to carry out. Acute hiPSC-based in vitro experiments can shed light on the elementary effects of psychotropic medications, which will increase our insight into the interaction of these molecules with viable human neurons. However, chronic treatment effects presumably elicit a chain of complex cellular events that are difficult to tease apart, therefore it must be kept in mind that in vitro experiments do not directly model neuropharmacological treatment; instead, they mimic the elementary mechanisms that occur in patients treated with neuropharmacological medication. Also, most patients usually receive a combination of drugs at different, individually titrated doses which are clinically meaningful, however it is very difficult to model this in vitro.

Chronic treatment of hiPSC-derived neurons has been attempted only in relatively few studies. Odawara et al. (2016) successfully maintained hiPSC-derived cortical neurons for up to one year and were able to demonstrate basic pharmacological properties and receptor profiles of the emerging mature neuronal networks (Odawara et al., 2016). Proconvulsants were observed to induce synchronised burst firing, which could be reversed by treatment with the anticonvulsant phenytoin. Grunwald et al. (2019) attempted to combine acute and chronic antipsychotic effects, investigating the effects of haloperidol, olanzapine and clozapine on neurite outgrowth dynamics, and the long-term effect of clozapine on gene expression (Grunwald et al., 2019). From their study, they were able to conclude that neurite outgrowth may not be a suitable functional readout for evaluating antipsychotic drug effects due to neurotoxicity. Overall, we can conclude that there is a scarcity of studies looking at the long-term effects of psychotropic medications, therefore such studies are necessary in the future.

### 4.6 Understanding and translating drug doses from bedside to bench to and vice versa

In a clinical setting, drug doses are determined specifically based on the label and recommended drug doses, and clinical parameters such as body weight, concomitant medication, and side-effect profiles. The basic method for clinical dose-finding is the gradual titration of doses from lower to higher. These practices are difficult to model in vitro, and other strategies should be used to identify optimal dosages for hiPSC-derived neurons. Peripheral drug and metabolite concentrations are available in most clinical centres, but these do not always easily translate to in vitro concentrations, due to blood-brain barrier effects and other pharmacokinetic and/or pharmacogenomics factors. Currently, most hiPSC-based studies attempt to model neuropharmacological effects by using multiple doses for in vitro experiments within a reasonable range, for example concentrations between 1 and 10 ​μM. Animal studies allow the possibility to obtain direct brain tissue concentrations of compounds, offering additional information regarding the similarity of in vitro and in vivo dosing.

As already discussed in this paper, several hiPSC-based studies have taken advantage of subtyping patient cohorts based on clinical response to a class of drugs. The rationale of such studies is that patient response to treatment is informative about a valid pharmacodynamic reaction, whereas patient non-response can have many reasons, such as pharmacokinetics, metabolism, or an insufficient pharmacodynamic effect. hiPSC lines derived from clinical responders increase the likelihood of observing a cellular phenotype in vitro. Mertens et al. (2016) generated hiPSC-derived dentate gyrus granule cells from bipolar disorder patients who responded clinically to lithium treatment, the gold standard mood stabiliser medication (Mertens et al., 2016). They observed that under baseline conditions, these cells were hyperexcitable, but that this disease phenotype could be rescued by in vitro lithium treatment. More recently, Vadodaria et al. (2019a and 2019b) generated hiPSC-derived serotonergic neurons from three patients with depression who positively responded to SSRIs treatments, and from three patients who were non-responders/non-remitters. Neurons from SSRI non-responders displayed longer total neurite length, more branch points, and hyperactivity in response to serotonin (Vadodaria et al., 2019a, 2019b). Despite the promise of performing hiPSC-based pharmacological studies on patient treatment response, it is important to note that poor adherence to treatment by patients could be a strong confounding variable, and results should always be interpreted with caution.

### 4.7 Drug screening assays for hiPSC-based studies: primary and secondary

In pharmacological research primary drug screens are usually performed by means of high-throughput screening, or utilise previous positive results to decrease the number of testable compounds, a process called focused screening. Primary screens are often carried out against targets without a cellular background, or simplified cellular model systems, such as target proteins expressed in tumour cells. These investigations are followed up by secondary drug screens, which usually involve dose-response curves and functional assays for the previously identified hits. While primary screens are considered a simple system, secondary drug screens involve more complex model systems.

Given the well-known complexity and labour-intensiveness of hiPSC-based in vitro studies, the authors believe that drug screening assays using hiPSCs are currently only feasible for secondary studies. Primary studies are possible to perform using hiPSCs and can provide a relatively quick overview and selection of potential effective therapeutic agents in absence of already available therapeutic agents with a desired effect. However the interpretation of the huge datasets would be challenging. Differentiating neurons can react to new compound treatment in many ways simultaneously (e.g. changes in neurite outgrowth combined with electrophysiological activity), and these changes might have opposite directional effects. Therefore, net effects would be difficult to model. However, the development of high throughput screening assays for intermediate cell types could be a potential solution. For example, Readhead et al. (2018) employed the use of hiPSC-derived neuronal progenitor cells from SCZ patients to perform high throughput pharmacological screening, thereby avoiding the methodological hurdles of neuronal differentiation, and were able to identify drugs that could reverse post-mortem SZ-associated transcriptomic signatures.

## 5 Conclusions

In this consensus paper, we brought together experts in hiPSC-based NPD research to provide guidelines for researchers wishing to use hiPSCs for NPD disease modelling, with a particular focus on neuropharmacological studies. We have specifically discussed experimental design, with the aim of increasing data reproducibility and robustness, which is currently an area of concern in the field. This is further highlighted by the new regulation from the Food and Drug Administration (FDA) in the United States who have stipulated that animal testing is no longer required for human drug trials, potentially shifting the emphasis towards an increase in the use of PSC-based models in drug discovery studies. We hope that this consensus paper will provide much-needed recommendations for best practice in hiPSC research and will stimulate further discussions regarding the standardisation of minimal requirements for robust data, allowing hiPSC-based research to realise its full potential.

## Funding

This work was supported by the 10.13039/501100007871ECNP, iPSCs Platform for Neuropsychiatry 10.13039/501100007871ECNP Network. This work (King's College, London) was also supported by the 10.13039/501100000265Medical Research Council (MRC) Centre grant (MR/N026063/1 - AVC, DPS). DPS was supported by a 10.13039/501100000265MRC grant (MR/X004112/1) and funds from the 10.13039/100014370Simons Foundation Autism Research Initiative (SFARI). EG, CMYO and NMW work was supported by the 10.13039/501100006447University of Zurich (UZH) Funds and the Psychiatric 10.13039/501100009396University Hospital Zurich research Funds no. 8702 (*Fonds für wissenschaftliche Zwecke im Interesse der Heilung von psychischen Krankheiten*). Swedish funding to A.F. VR (2019-01498), Hjärnfonden (FO2019-0246, FO2021-0234). I.E. was supported by a MSCA EF Seal Of Excellence postdoctoral fellowship from the Strategy Group of EU Coordination in Sweden (2021-01834). SKS was supported by an IZKF Würzburg grant N440. RVM was supported by a 10.13039/100009670NARSAD Young Investigator grant from the Brain and Behaviour Research Foundation (#30587) and Postdoc Plus funding from the Graduate School of Life Sciences, University of Würzburg. GCZ was supported by a grant from the 10.13039/501100001659DFG (Project No. 413657723; Clinician Scientist-Program UNION CVD). FR was supported by a grant from the 10.13039/501100001659DFG (Clinician Scientist Program UNION-CVD). JR was supported by the TKP2021-EGA-25 Thematic Excellence Grant of the 10.13039/501100015269Hungarian Government. This work was also supported by the Netherlands Organ-on-Chip Initiative (NOCI), an NWO Gravitation project funded by the Ministry of Education, Culture and Science of the government of the Netherlands (024.003.001; to F.M.S.d.V. and S.A.K.), and by the ZonMW PSIDER program TAILORED (10250022110002; to F.M.S.d.V., S.A.K). European COST Action, CA16210 Maximizing Impact of research in NeuroDevelopmental DisorderS (MINDDS).

## Declaration of competing interest

The authors declare no competing interests.

## 6. Abbreviations

2D: 2 Dimensional
3D: 3 Dimensional
5-HT: 5-Hydroxytryptamine
5-HT2A: 5-Hydroxytryptamine 2A receptor
5-HT7: 5-Hydroxytryptamine 7 receptor
ADHD: Attention Deficit Hyperactivity Disorder
ASC: Autism Spectrum Conditions
BDNF: Brain Derived Neurotrophic Factor
cAMP: Cyclic Adenosine Monophosphate
*CHRFAM7A*: CHRNA7-FAM7A fusion protein
*CHRNA7*: Cholinergic Receptor Nicotinic Alpha 7 subunit
CNS: Central Nervous System
CNV: Copy Number Variation
CRISPR/Cas9: Clustered Regularly Interspaced Short Palindromic Repeats/CRISPR-associated protein 9
DAergic: Dopaminergic
DALYS: Disability-Adjusted Life-Years
DEG: Differentially Expressed Gene
*DISC1*: Disrupted-In-Schizophrenia-1
DSM-5: Diagnostic and Statistical Manual 5
EBiSC: European Bank for induced pluripotent Stem Cells
ED50: Effective dose 50
ES cells: Embryonic Stem cells
*FAM7A*: Family with sequence similarity 7
FISH: Fluorescence *in situ* Hybridization
GBD: Global Burden Disease
GCCP: Good Cell Culture Practice
GDP: Gross Domestic Product
*GDPR*: General Data Protection Regulation
GMP: Good Manufacturing Practice
GWAS: Genome Wide Association Studies
hESCs: Human Embryonic Stem Cells
hiPSCs: Human induced Pluripotent Stem Cells
HNK: (6R-2R)-hydoxynorketamine
IC50: Inhibitory concentration 50
ICD: International Classification of Diseases
ISSCR: International Society for Stem Cell Research
Kb: Kilobase
lncRNA *XIST*: Long non-coding RNA X-Inactive Specific Transcript
MDD: Major Depressive Disorder
MoA: Mechanism of Action
mTOR: Mechanistic Target Of Rapamycin
NGN2: Neurogenin2
NPDs: NeuroPsychiatric Disorders
*PARK2*: Parkison protein 2
PCA: Principal Component Analysis
PCR: Polymerase Chain Reaction
PDE4: Phosphodiesterase 4
PK-PD: Pharmacokinetic – Pharmacodynamic
PRS: Polygenic Risk Scores
PSCs: Pluripotent Stem Cells
PTSD: Post-Traumatic Stress Disorder
QC: Quality Control
RNA-seq: RiboNucleic Acid sequencing
RUV: Remove Unwanted Variation
SABV: Sex As a Biological Variable
SNP: Single Nucleotide Polymorphism
SSRI: Selective Serotonin Reuptake Inhibitors
SZ: Schizophrenia
WAIS: Wechsler Adult Intelligence Scale
WHO: World Health Organisation
WISC: Wechsler Intelligence Scale for Children
Xa: X active
XCI: X-Chromosome Inactivation
Xi: X inactive
XiE: Xi Erosion
